# Harnessing RNA sequencing for global, unbiased evaluation of two new adjuvants for dendritic-cell immunotherapy

**DOI:** 10.18632/oncotarget.15190

**Published:** 2017-02-08

**Authors:** Till S.M. Mathan, Johannes Textor, Annette E. Sköld, Inge Reinieren-Beeren, Tom van Oorschot, Mareke Brüning, Carl G. Figdor, Sonja I. Buschow, Ghaith Bakdash, I. Jolanda M. de Vries

**Affiliations:** ^1^ Department of Tumor Immunology, Radboud Institute for Molecular Life Sciences, Radboud University Medical Centre, Nijmegen, The Netherlands; ^2^ Department of Oncology and Pathology, Karolinska University Hospital Solna, Karolinska Institute, Stockholm, Sweden; ^3^ Miltenyi Biotec GmbH, Bergisch Gladbach, Germany; ^4^ Department of Gastroenterology and Hepatology, Erasmus MC-University Medical Center, Rotterdam, The Netherlands; ^5^ Department of Medical Oncology, Radboud University Medical Centre, Nijmegen, The Netherlands

**Keywords:** dendritic cells, immunotherapy, RNA sequencing, adjuvants, protamine-RNA, transcriptomics

## Abstract

Effective stimulation of immune cells is crucial for the success of cancer immunotherapies. Current approaches to evaluate the efficiency of stimuli are mainly defined by known flow cytometry-based cell activation or cell maturation markers. This method however does not give a complete overview of the achieved activation state and may leave important side effects unnoticed. Here, we used an unbiased RNA sequencing (RNA-seq)-based approach to compare the capacity of four clinical-grade dendritic cell (DC) activation stimuli used to prepare DC-vaccines composed of various types of DC subsets; the already clinically applied GM-CSF and Frühsommer meningoencephalitis (FSME) prophylactic vaccine and the novel clinical grade adjuvants protamine-RNA complexes (pRNA) and CpG-P. We found that GM-CSF and pRNA had similar effects on their target cells, whereas pRNA and CpG-P induced stronger type I interferon (IFN) expression than FSME. In general, the pathways most affected by all stimuli were related to immune activity and cell migration. GM-CSF stimulation, however, also induced a significant increase of genes related to nonsense-mediated decay, indicating a possible deleterious effect of this stimulus. Taken together, the two novel stimuli appear to be promising alternatives. Our study demonstrates how RNA-seq based investigation of changes in a large number of genes and gene groups can be exploited for fast and unbiased, global evaluation of clinical-grade stimuli, as opposed to the general limited evaluation of a pre-specified set of genes, by which one might miss important biological effects that are detrimental for vaccine efficacy.

## INTRODUCTION

Antigen presenting cells, such as Dendritic cells (DCs), play a central role in many immunotherapies because of their ability to induce immune responses or to promote immune tolerance by interacting with CD4^+^ and CD8^+^ T cells. For T cell activation to occur, DCs need to mature and migrate to the lymph nodes. DC immunotherapies aim to strengthen antitumoral immune responses by boosting T cell activation [1, 2]. In such therapies, DCs are isolated, activated and loaded with tumor antigen and then given back to the patient. Vaccine DCs are anticipated to promote antitumor responses by presenting tumor antigen in the context of costimulatory molecules and immune-stimulatory cytokines [3–7]. Upon activation, DCs upregulate costimulatory markers like CD40 and CD80, but also co-inhibitory markers, such as PD-L1, PD-L2, IL-10 and TGF-β, which are essential for the termination of an immune response. Expression of the right maturation markers and secretion of the right cytokines is thus important for vaccine success and these therefore need to be taken into account when selecting the optimal adjuvant for the activation of vaccine DCs.

We perform immunotherapies with naturally occurring DCs, namely CD1c^+^ myeloid dendritic cells (mDCs) and plasmacytoid dendritic cells (pDC) [8, 9]. These two subsets possess complementary phenotypes: they secrete different cytokines, express different pattern recognition receptors (PPR), and even take different, incompletely understood, migratory routes [10–14]. Mature plasmacytoid DCs respond to viruses and are known to produce large amounts of type I IFNs upon activation and may also induce T cell responses [15–20]. Their counterparts, the CD1c^+^ mDCs, respond to various microbial and fungal stimuli and induce Th1 responses via the production of IL-12 upon maturation [11, 21–23]. Successful activation of naïve CD8^+^ T cells to cytotoxic CD8^+^ lymphocytes (CTLs) is of high interest for immunotherapies, since CTLs specifically target the cancerous cells [24, 25]. In several clinical studies, the presence of CTLs was associated with a higher survival or increased clinical response [26–28]. Taken together, because both DC subsets are able to provoke a Th1 response, combining them may increase the efficacy of the antitumor immune response. Our clinical trials with either pDCs, stimulated by FSME (an inactivated tick borne encephalitis virus that most likely binds to TLR7/8), or with CD1c^+^ mDCs using GM-CSF (a growth factor that promotes myeloid cell maturation), highlighted the anti-tumor potential and positive clinical outcome in melanoma patients [8, 9].

The only available good manufacturing practice (GMP) grade TLR7/8 ligand is pRNA, a complex of protamine and mRNA, which most likely triggers TLR7/8, similar to FSME [29]. Sköld *et al*. have recently shown that pRNA activates CD1c^+^ mDCs and pDCs and induces them to release IL-12 and IFN-α, respectively, making it a promising candidate to use for vaccines containing both subsets [30]. These findings prompted us to further inspect the effect of pRNA on DC activation. Furthermore, we investigated another novel clinical grade pDC activation stimulus, CpG-P. CpG ODNs are short single-stranded DNA molecules containing unmethylated CpG dinucleotides and can be divided in different classes depending on their effects. CpG-C and CpG-P combine both effects of CpG-A and CpG-B, namely strong IFN-α release and strong maturation marker upregulation [31, 32].

In our study we obtained and analyzed RNA sequencing data of the two DC subsets using these new clinical grade adjuvants conditions to obtain a comprehensive and unbiased overview of the effect of each stimulus on the phenotype of the activated DCs. Focusing only on specific maturation markers and cytokines may lead to a loss of relevant findings. Recently, the relevance of implementing systems biology in vaccine research has been demonstrated by studying the effect of human adjuvants in animal models with transcriptome profiling [33–35]. Because of its unbiased nature, system vaccinology may help to understand which immunological processes are detrimental for vaccine success [36–38]. In the present study, we applied principal coordinate analysis (PCoA) and gene ontology (GO) analysis to evaluate the effect of each adjuvant on the whole cell rather than selected maturation markers and cytokines only. Using these approaches, we compared the previously used DC activation stimuli, GM-CSF for CD1c^+^mDCs and FSME for pDCs, with the novel stimuli, pRNA and CpG-P, and validated several of our findings with functional assays. Our data indicate that both pRNA and CpG-P are suitable clinical grade adjuvants for the activation of either both DC subsets together or pDCs alone.

## RESULTS

### RNA-seq gene expression levels are comparable with protein levels

**Figure 1:**
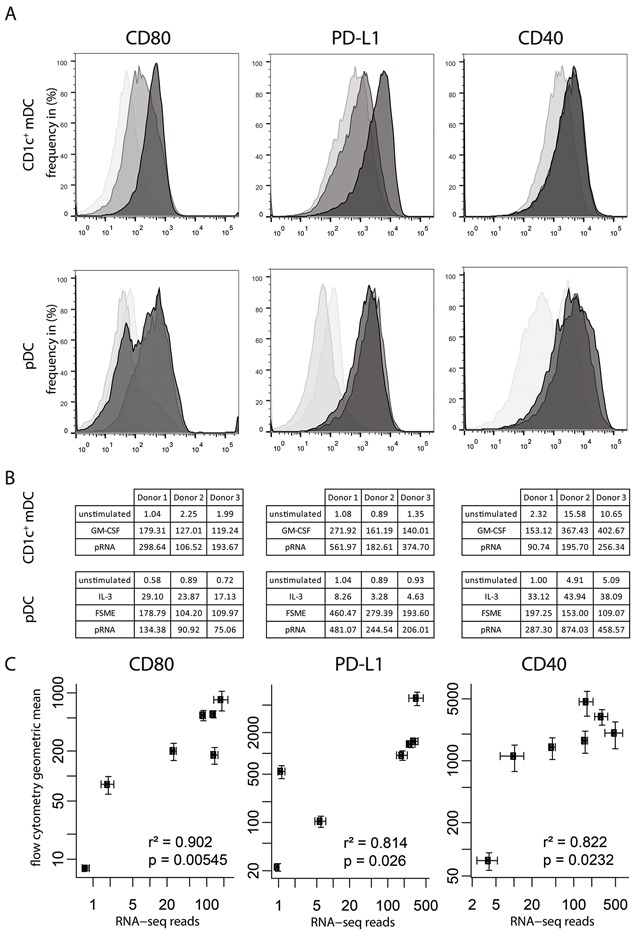
RNA-seq results represent protein levels **A**. Flow cytometry histograms for three maturation markers (CD80, PD-L1 and CD40) on pDCs and CD1c^+^ mDCs. For CD1c: Light grey represents unstimulated samples, dark grey represents GM-CSF samples, and black represents pRNA samples. For pDC: bright transparent grey represents the unstimulated samples, light grey represents the IL-3 samples, grey represents FSME and black represents pRNA samples. **B**. Gene expression levels of the 3 donors of the RNA-seq. **C**. Correlation plot of the RNA-seq counts/1 million reads set out against the geometric mean fluorescence intensity as measured by flow cytometry (protein level). Throughout this paper, error bars represent standard error of the mean.

To evaluate the transcriptome of the two DC subsets upon activation with different stimuli, we performed whole-cell RNA sequencing of magnetic bead isolated CD1c^+^ mDCs and pDCs of the same donors. To this aim, DC transcriptomes were analyzed directly after isolation or following 6 hours of stimulation with either IL-3 alone, FSME and IL-3 or pRNA and IL-3 (pDCs) or GM-CSF, pRNA (CD1c^+^ mDCs). First, we were interested in whether this approach would give comparable results as targeted flow cytometry evaluation of established DC maturation markers. Here we chose the markers CD80, PDL-1, and CD40 (Figure 1A–1C) as representatives for the maturation state of these DC subsets. CD80 is a co-stimulatory maturation marker also used in our clinical set up to determine the maturation state of the DCs [8, 39]. PD-L1 as a co-inhibitory and CD40 as an additional co-stimulatory marker were considered suitable candidates to complete the set, because both maturation markers are known to be expressed on both cell subsets [40]. Comparing the different conditions of pDCs with each other, we found that the overall pattern of RNA and protein expression was similar. The survival factor IL-3 showed no or very little effect on the expression of the maturation markers, except on CD40 (Figure 1C). FSME and pRNA, showed a similar upregulation of maturation markers CD80, PDL-1 and CD40 on pDCs. In CD1c^+^ mDCs, GM-CSF or pRNA caused an upregulation of all maturation markers, at both RNA and protein levels. However, there were some differences in the strength of the two stimuli: pRNA appeared to have a stronger affect on CD80 and PD-L1 RNA and protein expression. GM-CSF induced stronger CD40 RNA expression but this was not found at the protein level. In order to further delineate the relation between RNA-seq reads and protein expression levels, we performed a Spearman nonparametric correlation analysis with the RNA sequencing samples of the 3 donors and compared them to protein expression values of 6 different donors. All three maturation markers showed strong correlations between RNA and protein levels (CD80: r^2^=0.902, p=0.00545; PD-L1: r^2^=0.814, p=0.026; CD40: r^2^=0.822, p=0.0232). Altogether, RNA seq data well reflected flow cytometry data indicating the biological relevance of changes in RNA expression values. Furthermore, this first analysis suggests that pRNA is equally or even more potent than currently used clinical grade stimuli in the activation of CD1c^+^ mDCs and pDCs.

### Clinical grade stimuli have similar overall effects on both cell types

**Figure 2:**
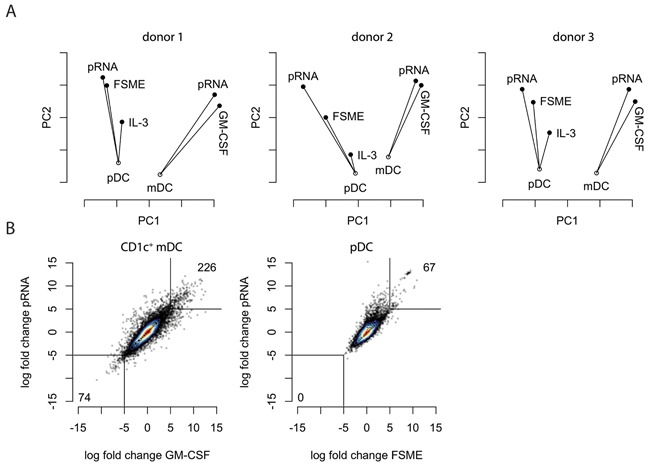
RNA-seq-based global assessment of DC responses to clinical stimuli **A**. Principal coordinates analysis (PCoA) of pDCs and CD1c^+^ mDCs was performed for each of the stimuli to compare their similarities in gene expression. Each point represents the transcriptome of the respective condition and the analysis was based on the first and second coordinate. On the x-axis the principle coordinate one (PCO1) is displayed, on the y-axis the principle coordinate two (PCO2). **B**. Correlation plots depicting the gene expression changes upon each stimulus for each subset. Each point represents a gene.

Having confirmed the validity of RNA-seq for evaluating DC maturation status, we were interested in how specific stimuli affect the entire DC transcriptome, rather than a small pre-defined set of maturation markers. Therefore, we applied principal coordinates analysis (PCoA) on the gene expression data of all DC types and conditions tested, separately per donor. The first two principal coordinates are shown in Figure 2A. The first coordinate (X axis) aligned roughly with the cell type (pDC versus CD1c^+^ mDC), whereas the second coordinate reflected maturation status. The overall picture was similar between the three donors, indicating good reproducibility of the RNA sequencing.

In both DC subsets the new stimulus, pRNA, showed a similar effect as the previously used stimuli FSME and GM-CSF. While the differences in effects between GM-CSF and pRNA, as measured by the first two principal coordinates, were very small for CD1c^+^ mDCs, the differences between stimuli were more pronounced on pDCs. Using the unstimulated pDCs as the reference point, the FSME sample and pRNA did align along the same axis, but pRNA was located further away from the unstimulated cells. This suggests that both stimuli affect the RNA expression of roughly the same genes, but that pRNA has an overall stronger effect on these genes. Furthermore, the data indicated that upon stimulation, the differences between the two DC phenotypes increased.

To directly compare the established and the novel stimuli for each of the two cell types, we correlated the fold-changes of each gene relative to unstimulated cells (for CD1c^+^ mDCS) or to IL3-treated cells (for pDCs) of existing and novel activation stimuli (Figure 2B). Both plots confirmed the overall similar effect of the existing and novel stimuli on the transcriptome we already observed by PCoA. In line with PCoA, a slight skewing of the pDC correlation plot towards pRNA indicated that indeed pRNA had a stronger effect than FSME on mostly the same set of genes. Interestingly, for pDCs more genes were highly upregulated (>10 fold) than highly downregulated by both stimuli, whereas for CD1c^+^ mDCs this difference was less pronounced (Figure 2B).

To find out which biologically coherent gene groups were most affected by the different stimuli, a gene ontology (GO) term analysis was performed (Table 1) on all significantly (p<0.05 after multiple testing correction with the Benjamini-Hochberger method) up- and downregulated genes of each subset. This qualitative method also provided insight in affected pathways and gene groups not directly connected to DC maturation. Overall, all stimuli most strongly affected immune response-related genes. However, GM-CSF treatment of CD1c^+^ mDCs also clearly affected a number of gene clusters that are not directly linked to immune response. Of note, one of these clusters was “nonsense-mediated decay” [41], pointing to a possible deleterious effect.

**Table 1:** Clinical stimuli affect similar gene clusters in pDCs but less so in CD1c^+^ mDCs

term	ontology	N genes	N up/down	- log_10 p-value
**CD1c^+^ mDC GM-CSF**				
cytosolic ribosome	CC	103	62	25.4
extracellular region part	CC	3752	755	22.5
SRP-dependent cotranslational protein targeting to membrane	BP	108	60	22.1
cotranslational protein targeting to membrane	BP	110	60	21.5
nuclear-transcribed mRNA catabolic process, nonsense-mediated decay	BP	120	63	21.4
immune system process	BP	2576	547	20.6
protein targeting to ER	BP	114	60	20.5
extracellular region	CC	4482	862	20.0
establishment of protein localization to endoplasmic reticulum	BP	118	60	19.5
regulation of multicellular organismal process	BP	2403	507	18.4
**CD1c^+^ mDC pRNA**				
immune system process	BP	2576	578	21.3
regulation of cell migration	BP	593	177	17.8
cell surface receptor signaling pathway	BP	2677	580	17.8
regulation of cell motility	BP	628	184	17.6
immune response	BP	1626	383	17.2
regulation of cellular component movement	BP	704	199	17.1
inflammatory response	BP	616	180	17.1
extracellular region part	CC	3752	764	16.6
response to organic substance	BP	2828	601	16.5
regulation of locomotion	BP	690	194	16.4
**pDC FSME**				
immune response	BP	1626	127	26.9
immune system process	BP	2576	165	25.9
response to type I interferon	BP	85	30	24.7
response to virus	BP	403	57	23.7
type I interferon signaling pathway	BP	84	29	23.6
cellular response to type I interferon	BP	84	29	23.6
defense response to virus	BP	325	51	23.3
response to biotic stimulus	BP	904	85	22.7
response to external biotic stimulus	BP	869	82	22.0
response to other organism	BP	869	82	22.0
**pDC pRNA**				
response to virus	BP	403	73	24.4
immune response	BP	1626	166	24.3
immune system process	BP	2576	222	23.1
cytokine activity	MF	218	51	22.3
response to type I interferon	BP	85	33	22.2
defense response to virus	BP	325	62	22.0
response to external biotic stimulus	BP	869	107	21.4
response to other organism	BP	869	107	21.4
type I interferon signaling pathway	BP	84	32	21.2
cellular response to type I interferon	BP	84	32	21.2

Changes in gene expression clusters were detected by performing a GO term analysis. Of each stimulus the top 10 most differential expressed gene clusters were selected. The description for each gene cluster is mentioned in the columns. The ontology defines the sub-ontology: molecular function (MF), cellular component (CC) and biological process (BP). *N genes* stands for the total amount of genes for this gene cluster and *N up/down* for the number of genes that changed their expression values upon the stimulus. The table is sorted on the –log_10_ p-value, which can be found in the column on the very right.

Together, the whole-transcriptome analysis indicated that pRNA had similar effects on both cell types as the existing cell type-specific stimuli. However, pRNA appeared to have an overall stronger maturation effect on pDCs and a similar effect on CD1c^+^ mDCs compared to the previously used stimuli. Importantly, unlike GM-CSF, pRNA did not have any obvious negative effects on CD1c^+^ mDCs.

### Discrepancies between pRNA stimulated pDCs and CD1c^+^ mDCs

**Figure 3:**
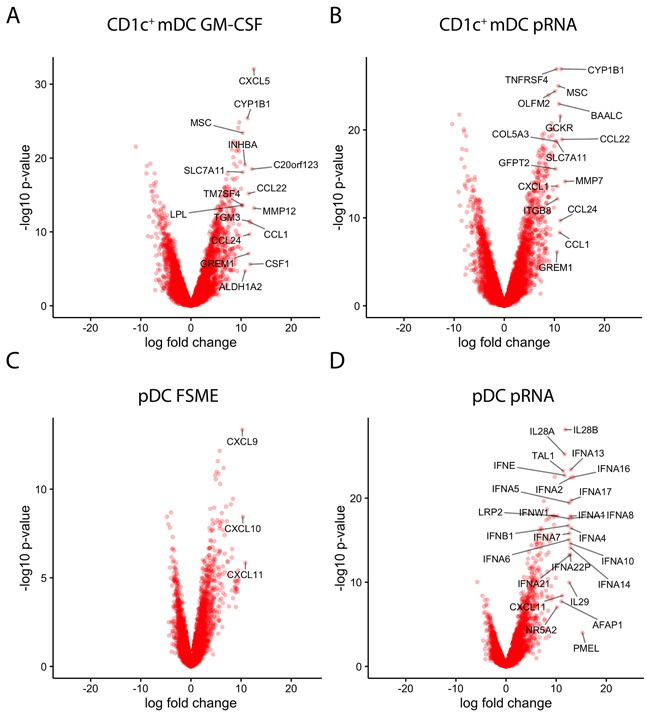
Stimuli effects on individual genes **A-D**. Volcano plots representing the gene expression changes (x-axis: log fold) together with the statistical significance (y-axis: -10log p-value). Each condition was compared to the respective unstimulated cell type. Genes with a log fold change of more than 10 were labeled with the gene name. A) GM-CSF stimulated CD1c^+^ mDC; B) pRNA stimulated CD1c^+^ mDCs; C) FSME stimulated pDCs; D) pRNA stimulated pDCs.

Besides the overall similarities among used stimuli, we also observed differences between the effects of the existing and novel stimuli on each cell subset. A limitation of the GO term analysis is that it does not take into account the magnitude of the expression changes and therefore no conclusions on these can be drawn. Therefore, as a complementary approach, we generated Volcano plots of the overall fold-changes and p-values for each gene based on the combined data from all 3 donors. These plots show that pRNA in CD1c^+^ mDCs caused a strong upregulation of cytokines and migration-related genes (Figure 3). In CD1c^+^ mDCs stimulated with GM-CSF, several chemokines (CXCL5, CCL1 CSF1, CCL24 and CCL22) were upregulated 12-13 fold. Upon activation with pRNA, chemokines were once again found among the most upregulated genes. Except for CXCL5 and CCL17, these genes were upregulated to a very similar extent as upon GM-CSF stimulation. Comparing the chemokines with each other, the fold changes were almost on the same level between the two stimuli (Figure 3A and 3B).

**Figure 4:**
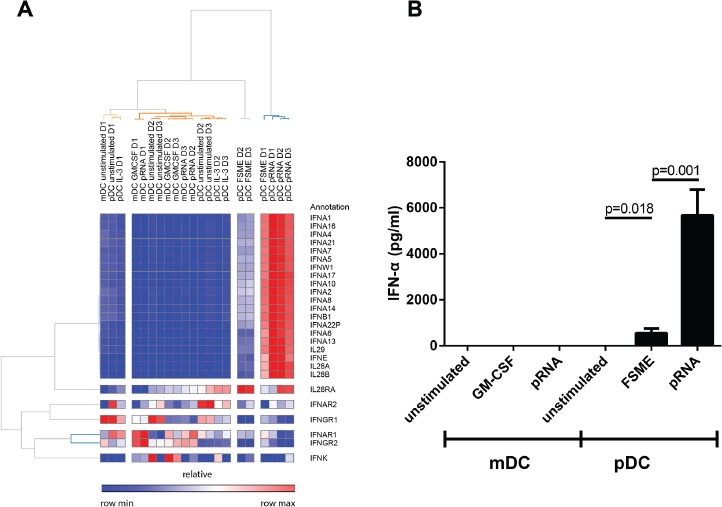
High type I/III interferon production of pDCs upon pRNA stimulation **A**. Heat map of the type I/III interferon genes. Red colour represents high expression and blue low expression. **B**. Type I interferon levels on protein level measured in the supernatant of the stimulated cells after an overnight incubation (n=7).

In pDCs, multiple genes related to type I and III interferons (IFN-α and IFN-λ) were found among the most strongly and significantly upregulated genes. While pRNA lead to a significant increase of the transcription of various subtypes of IFN-α and IFN-λ (IL28/ IL29), FSME upregulated the chemokines CXCL9, CXCL10 and CXCL11 when compared to unstimulated pDCs. As already indicated by PCoA and correlation plots (Figure 2), the overall extent of gene expression increase upon FSME was less than upon pRNA stimulation. For instance, whereas FSME caused a >10-fold increase for only 3 genes, pRNA did so for 26 genes. Several IFN-α subtypes showed 13-fold increases when stimulated with pRNA, compared to 9- to 10-fold for FSME (Figure 3C and 3D). As expected the difference between DC subtypes was most prominent for type I IFNs, pRNA induced no IFN-α production in CD1c^+^ mDCs in contrast to high levels in pDCs. In pDCs, all 13 type I IFN genes were upregulated upon pRNA stimulation in all 3 donors (Figure 4A). We confirmed this observation at the protein level in 7 donors and detected a significantly higher (10-fold, p<0.001) release of IFN-α upon pRNA compared to FSME stimulation (Figure 4B).

**Figure 5:**
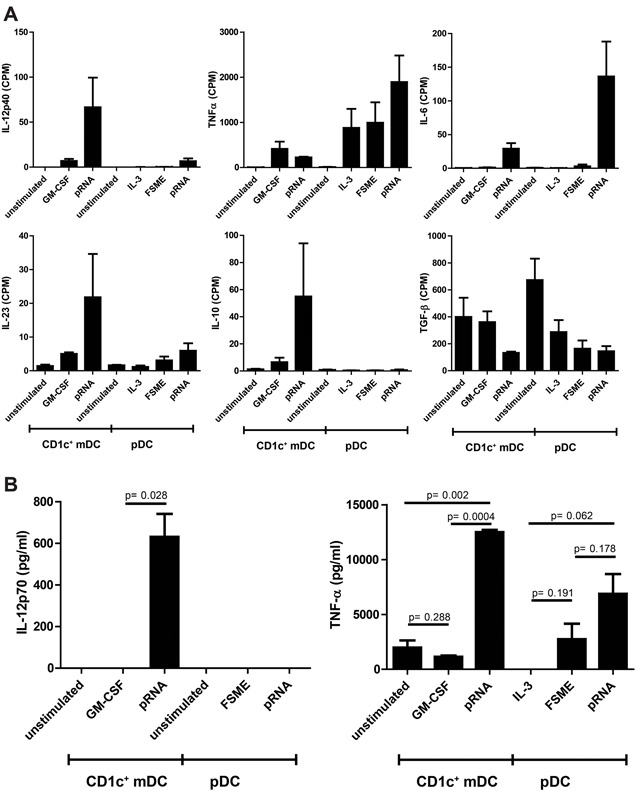
Pro-and anti-inflammatory cytokine release by CD1c^+^ mDCs and pDCs **A**. RNA expression values of several pro-inflammatory (IL-12p70, TNF-α, IL-23 and IL6) and anti-inflammatory cytokines (IL-10 and TGF-β) of pDCs and CD1c^+^ mDCs. **B**. IL-12p70 and TNF-α levels on protein level were measured in the supernatant of the stimulated cells after overnight incubation (n=3).

To assess the effect of pRNA on CD1c^+^ mDC, we assessed additional immunostimulatory cytokines that are known to be relevant for this subset. Based on the RNA-seq data, the gene expression of the immunostimulatory cytokines IL-12p40, IL-23 and IL-6 increased significantly upon pRNA stimulation (Figure 5A). TNF-α, another immunostimulatory cytokine, was increased on both pDCs and (less strongly) on CD1c^+^ mDCs. Additionally, the transcription of the immunoinhibitory cytokine IL-10 increased by pRNA stimulation compared to the unstimulated and GM-CSF-stimulated samples. We chose IL12p70 and TNF-α as representing cytokines to confirm the RNA-seq results on the protein level (Figure 5B). IL-12p70 release showed the same pattern as the RNA-seq counts, whereas for TNF-α release the results differed slightly from the RNA-seq data: On protein level, pRNA was the most effective stimulus for TNF-α production on both cell types, whereas the RNA-seq counts suggested GM-CSF as a slightly more potent TNF-α stimulus.

Taken together, our results suggest pRNA to be a potent activation stimulus for pDCs as well as CD1c^+^ mDCs, reflected by a strong upregulation of type I IFN and chemokines.

### CpG-P as a clinical grade stimulus for pDCs

**Figure 6:**
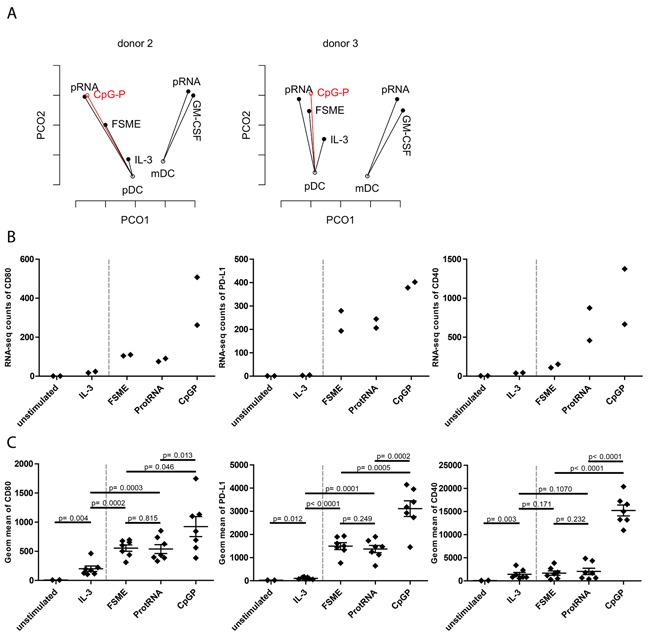
Assessment of CpG-P as a clinical-grade stimulus **A**. PCoA of pDCs of donor 2 and 3 with CpG-P data included in the figure. Each point represents the transcriptome of the respective condition and the analysis was based on the first and second coordinate. **B**. Upregulation of the maturation markers on pDCs at RNA-seq level upon indicated stimuli (n=2). Upregulation of the maturation markers on pDCs at protein level upon the indicated stimuli (n=7).

Finally, we investigated CpG-P, a novel clinical grade TLR9 agonist, as a pDC-specific activator. These synthetic oligonucleotides mimic the effect of bacterial DNA and therefore may support Th1 responses. We used RNA-seq on pDCs from two donors to evaluate the effect of CpG-P on pDCs and validated our findings with additional functional assays on protein level. For that purpose, we incorporated the CpG-P stimulated pDC samples into the PCoA analysis and evaluated their positions. The CpG-P samples appeared to be similar to pRNA in Donor 2 and close to both pRNA and FSME in Donor 3 regarding overall gene expression levels (Figure 6A). These results suggested that CpG-P is a strong activation stimulus for pDCs. To get a more detailed view of the changes in gene expression of DC-specific genes, we focused on the DC maturation markers shown in Figure 1. CpG-P induced a stronger upregulation of transcripts and proteins of all 3 maturation markers than FSME and pRNA (Figure 6B & 6C). In addition to the maturation markers, the release of type I IFN was strongly upregulated by CpG-P (Supplementary Figure 1). Altogether, our RNA-seq method suggests CpG-P as a strong adjuvant for pDCs.

## DISCUSSION

In this study, we aimed to evaluate the efficiency of two novel clinical grade adjuvants in a comprehensive and unbiased manner. We therefore used RNA-seq to evaluate the effects of each stimulus on total gene expression in two DC subsets of interest for immunotherapy. This approach allowed us to directly and extensively compare the effects of different adjuvants on DC phenotypes, beyond conventional maturation markers [40]. With our approach we translated changes on the whole-transcriptome level to possibly functionally relevant effects. Firstly, the results revealed the effect of different stimuli in each donor and allowed us to draw conclusions based on these patterns. Secondly, the similarity of the results in all three donors indicated good reproducibility of this method. Importantly, we were able to confirm RNA-seq expression changes regarding maturation marker expression and cytokine release on protein level and therefore validate the expression differences we observed in the transcriptome. This allowed us to draw conclusions about the efficacy of the tested clinical grade stimuli.

The PCoA analysis generated a useful initial summary of the whole-transcriptome data into a simplified representation that nevertheless revealed striking relations between the individual datasets. Importantly, this approach allowed for an overall assessment of the “strength” of each stimulus. This analysis suggested that pRNA is potentially a stronger pDC stimulus than FSME, with qualitatively similar effects as indicated by the GO analysis (Table 1). We demonstrated in a previous study that pRNA–induced activation of CD1c^+^ mDCs leads to significantly higher IL12p70 release and a similar TNF-α production as R848 and Poly IC, combined with lower immunosuppressive IL-10 production [30]. The robustness of these findings was corroborated by our functional assays on protein level. Additionally, based on PCoA, CpG-P was identified as a potentially strong stimulus for pDCs. Our functional assays confirmed this finding by showing that CpG-P not only upregulated the maturation markers significantly more than the other stimuli, it also led to a very high release of immunostimulatory cytokines such as IFN-α. Since type I IFNs are known to play important roles in T cell activation, these findings suggest that CpG-P could prove to be a potent pDC stimulus in immune therapies [42]. Interestingly, the transcriptome changes by CpG-P stimulation are very similar to the ones induced by pRNA, even though they stimulate different TLRs.

GM-CSF is widely used to differentiate *in vitro* monocytes into DCs and its high levels are connected to higher numbers of moDCs *in vivo* [43–47]. However, GM-CSF has been shown to inhibit pDC development via IRF8 [48]. GO-analysis of the effect of GM-CSF on CD1c^+^ mDC indicated that upon GM-CSF stimulation, a significant number of genes belonging to the GO term “nuclear-transcribed mRNA catabolic process, nonsense-mediated decay (NMD)” were differentially expressed. This NMD pathway activates the destruction of mRNAs containing premature stop codons, which has negative side effects [49, 50]. How this pathway affects the maturation process of CD1c^+^ mDCs and their functionality has not been reported yet, but it may be involved in inflammatory processes [51]. An unexpected side-effect like this would not have been found by using FACS analysis on an *a priori* defined, limited set of genes. This should be considered when GM-CSF is clinically applied, as inducing this gene cluster may have a negative impact on CD1c^+^ mDC function.

Other studies have already applied RNA-seq to characterize DCs upon stimulation with antigen. For example, two recent studies have applied single cell RNA-seq in a non-clinical context to identify differences between DC subsets and the effect of pathogenic stimuli on these cells [52, 53]. Furthermore, there is considerable interest to apply RNA-seq in the clinic for diagnostic purposes, with several applications already underway [54]. Complementing these efforts, our study highlights the usefulness of RNA-seq based approaches for the design of clinical therapies, as it allows to determine the effects of certain stimuli on the target cells. In addition to RNA-seq, proteomics is another powerful unbiased approach that can be used to study the differences between DC subsets [55].

Taken together, a whole-transcriptome approach was used to analyze the effect of clinical grade stimuli on human DCs. This method provides a global, unbiased overview of how cells react to certain stimuli. In our case, we could confirm pRNA as a potent stimulus for both pDCs and CD1c^+^ mDCs and introduce CpG-P as a new clinical grade stimulus for pDCs. *In vitro*, both new stimuli outperformed or had clear advantages over the existing stimuli.

## MATERIALS AND METHODS

### Cell isolation and culture

For functional assays, DCs were isolated from buffy coats of healthy volunteers (Sanquin, Nijmegen, the Netherlands) after obtaining written informed consent per the Declaration of Helsinki and according to institutional guidelines. For RNA-seq measurements, cells were obtained from aphaeresis of 3 different donors. Peripheral blood mononuclear cells (PBMCs) were isolated by using Ficoll density centrifugation (Lymphoprep; Axis-Shield PoC AS, Oslo, Norway). CD1c isolation kit of Miltenyi Biotec (Bergisch-Gladbach, Germany) was used to obtain CD1c^+^ mDCs, by following manufacturer's instructions. Subsequently, monocytes were depleted by either plastic adhesion, or by the use of CD14 microbeads (Miltenyi Biotec). Next, pDCs were purified by positive selection using anti–BDCA-4–conjugated magnetic microbeads (Miltenyi Biotec). DCs were cultured in X-VIVO-15 medium (Lonza, Basel, Switzerland) supplemented with 2% human serum (Sanquin). DCs were stimulated with: FSME (5%; Baxter AG, Vienna), pRNA (15μg/ml), CpG-P (5μg/ml; Miltenyi Biotech, Germany), GM-CSF (800 U/ml; (Cellgenix, Freiburg, Germany). pRNA complexes were prepared fresh 5-10 minutes before adding to the cell culture. pDCs were cultured with 10 ng/mL IL-3 (Cellgenix, Freiburg, Germany) as a survival factor in addition to the stimuli.

### Protamine-RNA complexesp

RNA complexes were made freshly before adding to the cells. Protamine (protaminehydrochloride MPH 5000 IE/ml; Meda Pharma BV Amstelveen, the Netherlands) was diluted to 0.5 mg/ml in RNase free water and mixed with 2-kbp-long single-stranded mRNA (coding for gp100). It was extensively mixed and incubated for 5-10 minutes at room temperature, before added to the cells.

### FACS phenotyping

The phenotype of pDC and CD1c^+^ mDC populations was determined by flow cytometry. DC purity was assessed by double staining CD11c^+^/CD1c^+^ for CD1c^+^mDCs (above 95%) and BDCA2/CD123 for pDCs (above 95%; all Miltenyi Biotec) [56]. The following primary monoclonal antibodies (mAbs) were used to determine the maturation state of the DCs: anti–CD80-APC, anti–PD-L1-APC (all BD Bioscience Pharmingen, San Jose, CA); and anti–CD40-PE (Beckman Coulter, Marseille, France). Measurements were performed on FACSCalibur and FACSVerse flowcytometers (BD).

### Cytokine detection

Supernatant was taken from each sample after overnight incubation and analyzed with standard sandwich ELISAs detecting IFN-α (Bender Medsystems, Vienna, Austria).

### RNA sequencing

CD1c^+^ mDCs and pDCs were isolated as described above and total RNA was extracted using Trizol (Invitrogen, MA, USA), following the standard protocol. The quality control of the isolated RNA (concentration, RIN, 28S/18S and size) was performed with Agilent 2100 Bioanalyzer (Agilent Technologies, Santa Clara, USA). RNA sequencing and read alignment were performed by BGI TECH SOLUTIONS (Hong Kong). Reads were aligned to human genome version 19. RNA sequencing data is deposited at the Gene Expression Omnibus (GEO; accession number: GSE89442).

### Hierachical clustering

Data was transformed to log values for performing hierarchical clustering analysis using the standard settings of the GENE-E software (Broad institute, Cambrige, MA; http://www.broadinstitute.org/cancer/software/GENE-E/index.html).

### Statistical analysis

Data was analyzed using the R platform for statistical computing. Specifically, the package “edgeR”, version 3.12, was used for whole-transcriptome principal coordinates analysis (using the “plotMDS” command), differential expression analysis, and GO term analysis [57]. Differential expression was determined by fitting a generalized linear model using the “glmFit” command, and significance was determined using the likelihood ratio test provided by the “glmLRT” command [58]. The R scripts used to perform the data analysis are available as Supporting Information for this manuscript.

### Abbreviations

pDC, plasmacytoid dendritic cells; mDC, myeloid dendritic cells; RNA-seq, RNA sequencing; FSME, Frühsommer meningoencephalitis; GM- CSF, granulocyte-macrophage colony-stimulating factor; pRNA, protamine-RNA; CpG-P, CpG oligodeoxynucleotides; TLR, toll like receptor.

## SUPPLEMENTARY MATERIALS FIGURES AND TABLES




