# Supplementary material for: Harnessing RNA sequencing for global, unbiased evaluation of two new adjuvants for dendritic-cell immunotherapy
Source: Oncotarget. 2017 Feb 8;8(12):19879–93. doi: 10.18632/oncotarget.15190 (PMC5386730; doi:10.18632/oncotarget.15190)
Supplement: Supplementary file 1 [file oncotarget-08-19879-s001.pdf]

Harnessing RNA sequencing for global, unbiased evaluation of two new adjuvants for dendritic-cell immunotherapy

SUPPLEMENTARY FIGURE

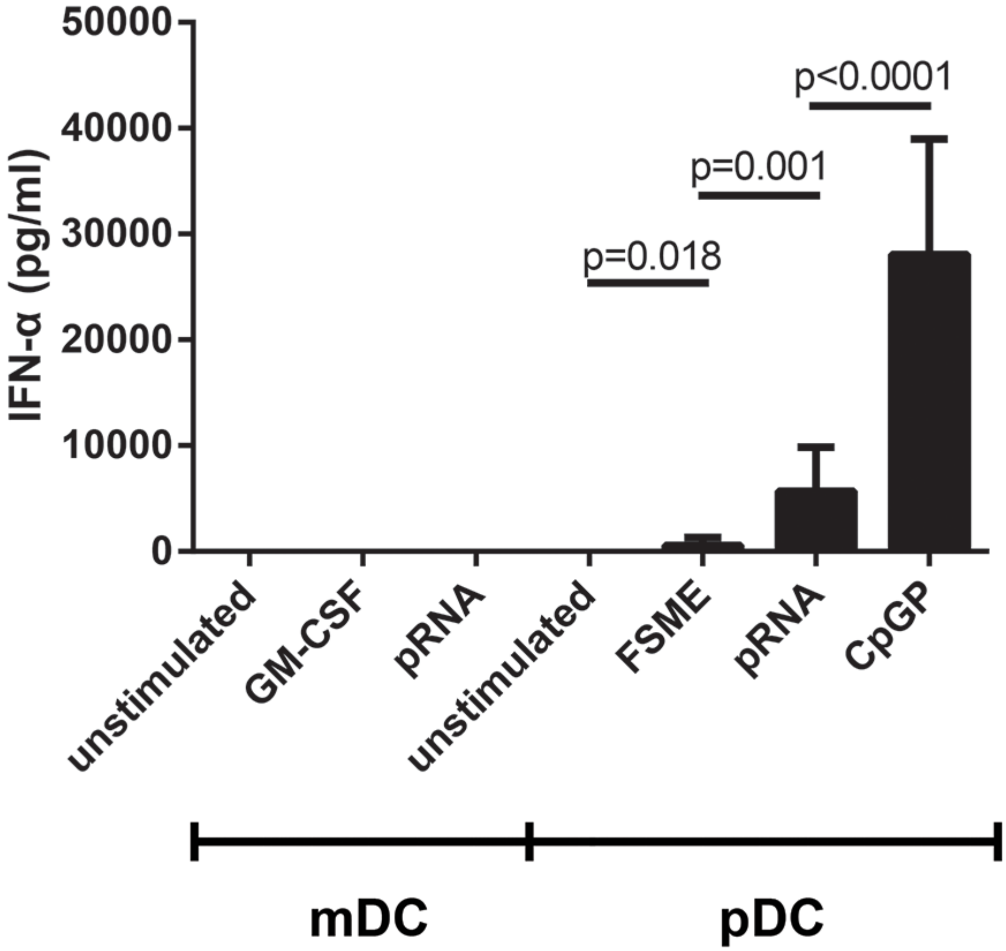

Supplementary Figure 1: High type I interferon production of pDCs upon CpG-P stimulation. A. Type I interferon release on protein level as measured in the supernatant of DCs after overnight incubation with the indicated stimuli (n=7).
